# Climate communication for biologists: When a picture can tell a thousand words

**DOI:** 10.1371/journal.pbio.2006004

**Published:** 2018-10-09

**Authors:** Stephan Lewandowsky, Lorraine Whitmarsh

**Affiliations:** 1 University of Bristol, Bristol, United Kingdom; 2 School of Psychological Science, University of Western Australia, Crawley, Western Australia, Australia; 3 School of Psychology, Cardiff University, Cardiff, United Kingdom

## Abstract

Pictures often tell a story better than the proverbial 1,000 words. However, in connection with climate change, many pictures can be highly misleading, for example, when a snowball is used to ridicule the notion of global warming or when a picture of a dead crop is supposed to alert people to climate change. We differentiate between such inappropriate pictures and those that can be used legitimately because they capture long-term trends. For example, photos of a glacier’s retreat are legitimate indicators of the long-term mass balance loss that is observed for the vast majority of glaciers around the world.

This Perspective is part of the *Confronting Climate Change in the Age of Denial Collection*.

On 26 February 2015, Senator James Inhofe (R–Oklahoma) famously brought a snowball to the floor of the United States Senate in an apparent attempt to disprove global warming and to question the fact that 2014 had been the hottest year on record at the time. (The three years since then, 2015, 2016, and 2017, have all been hotter than 2014.) Although scientifically ludicrous, Senator Inhofe’s snowball stunt may have resonated with the public, given that people are known to be readily influenced by anecdotes, images, and experiences. For example, people’s acceptance of climate change is increased after they need to process heat-related words such as boil, burn, sweat, and equator, as opposed to neutral words [1]. Similarly, people’s acceptance of climate change is a function of perceived temperature on the day [2], and even US newspaper editorials appear slanted in a direction determined by seasonal variation; hotter summers are followed by more editorials that endorse the scientific consensus position on global warming than relatively cooler summers [3].

This basic human tendency to rely on anecdotes, stories, and recent experiences presents a particular dilemma in relation to climate change. Perhaps more than most other scientific facts, the evidence for climate change is based on statistical analyses of innumerable observations that are dispersed across time and space. It takes up to 17 years of data to reliably detect a warming trend [4], and the large variability of the weather that is superimposed on that inexorable trend always provides an opportunity to point to some location on Earth that is experiencing record-breaking cold or snowfall, thereby providing an anecdote that, in people’s minds, may overpower the overwhelming scientific evidence that the globe is warming. Depending on the communicator’s intent, anecdotes can also be exploited to underscore the seriousness of climate change, for example, by pointing to drought-stricken cornfields, to parched landscapes, or—perhaps most iconic of all—to starving polar bears. Although such anecdotes and images avoid being in conflict with the scientific consensus, they may also be misleading because not all droughts and parched landscapes can be unambiguously attributed to climate change. Likewise, not all starving polar bears are starving because of the deterioration of their Arctic habitat. Although that deterioration and the consequent risk to the species’ welfare constitute a scientific consensus position [5], it does not follow that any particular polar bear in distress is a victim of climate change. The same holds true for any other species under threat from climate change; we can never be certain that an individual animal’s fate was sealed by climate change, no matter how great the risk to its species.

How can we resolve this conundrum? How can we legitimately use the anecdotes and images that we, as humans, find so alluring and convincing without risking scientific inaccuracy? The resolution lies in, first, understanding how people can become emotionally engaged with climate change and, second, in identifying legitimate triggers for that affect.

## Risk and affect

Studies from a range of areas, including risk perception, persuasion, and behavior change, highlight the importance of emotional engagement for motivating public response to societal issues [6]. Risk perception research, for example, shows that the “affect heuristic” is a powerful filter through which individuals assess the importance of risk issues [7]. Risk information is evaluated in light of how one feels about the issue in question, such that risks about activities we enjoy (e.g., drinking alcohol) are more likely to be downplayed than risks about activities we are less positive about (e.g., genetic manipulation [8]).

Where cognitive and affective assessments of risk diverge, it is affective assessments that tend to drive behavior [9]. This finding meshes well with the idea that there are two modes or “systems” of information processing: The “experiential” System 1 is assumed to be fast, emotional, and intuitive. The “analytic” System 2, by contrast, is slow and deliberative [6]. System 1 has evolved to keep us from harm, and it still dominates in everyday life. Although efficient and quick, System 1 can lead to biases and errors in judgement. For example, in the context of climate change, experiential processing leads us to underestimate the probability of extreme weather events (since they are rarely experienced) but to overestimate their reoccurrence when they do happen [10].

This highlights one of the challenges of engaging the nonexpert public with climate change. The issue is psychologically distant and lacks tangibility or relevance for most of us [11]. There is growing evidence that the lack of public engagement with climate change to date is at least in part due to a failure to communicate the issue in terms that resonate with individuals at a deeper and less distant level [12]. This distance can be overcome by the use of stories, images, and arts, which have been shown to engage the public at an emotional (“affective”) level with climate change [13,14].

Using such affective approaches to connect with audiences at an intuitive, visceral level (i.e., by engaging System 1) can reveal why people should care, especially if the images or stories demonstrate the significance of climate change for valued objects, places, or people [15]. Another approach relies on the arts: people who view a piece of art necessarily become more engaged with the object being depicted because they need to figure out what it means—especially when the art is abstract. Those moments of reflection, in turn, may open a window of opportunity for the contemplation of behavioral change [16].

However, not all emotional approaches are effective. Some past attempts to use visual imagery to communicate climate change have evoked negative emotions, such as fear, through conveying apocalyptic visions of the future. Unfortunately, these may actually demotivate audiences, triggering denial or apathy instead of engagement [17]. Similarly, some images that are iconic of climate change—such as a polar bear on a shrinking ice floe—may serve to reinforce the impression that climate change is distant and hence irrelevant [14].

## Triggering positive affect

Images or stories that demonstrate the significance of climate change for valued objects, places, or people—without evoking demotivating fear—can be effective triggers for public engagement. The need for affective engagement must, however, be balanced with informational content that accurately conveys the causes or impacts of climate change. If neither a snowball nor pictures of polar bears or parched landscapes are legitimate images, what are?

The crucial attribute of legitimate triggers is that they capture the long-term trend that characterizes climate change, rather than short-term phenomena or random events (commonly known as “weather”). Moreover, the triggers must be representative of a global pattern rather than a “cherry-picked” result. We illustrate this attribute with two examples involving the shrinking cryosphere and the implications of sea level rise.

**Fig 1:**
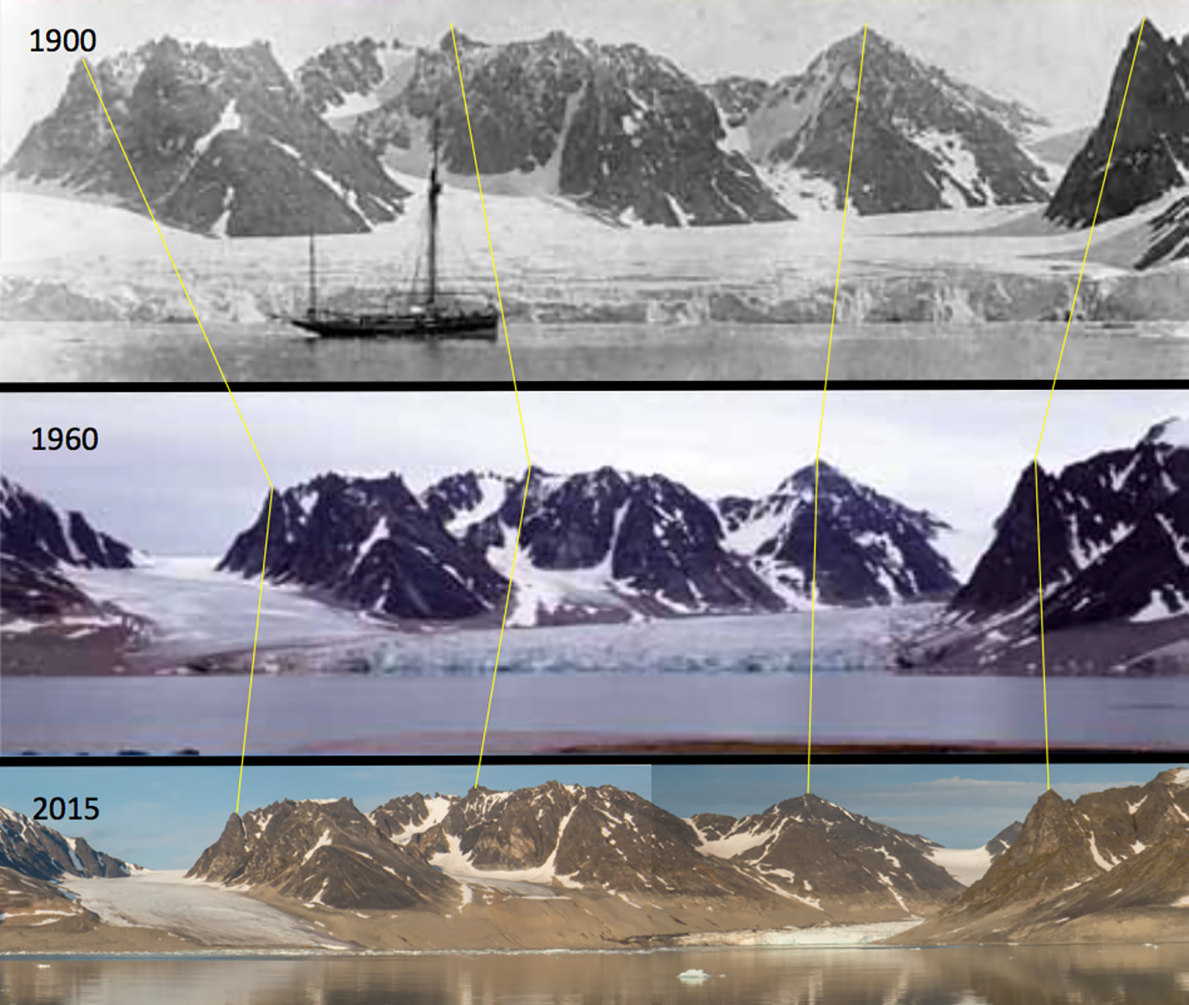
Retreating glacier on Svalbard. (Creative Commons license: https://commons.wikimedia.org/wiki/File:Glacier_decrease_on_Svalbard_in_the_years_1900-1960-2015.jpg).

Most glaciers around the world, and the Greenland and Antarctic ice caps, are shrinking because of anthropogenic climate change (e.g., [18]). Unlike snow fall (or snowballs), which are seasonal phenomena that reflect weather not climate, the extent of ice caps and glaciers are determined by climate. Each body of ice integrates across time, and its mass reflects the balance of accumulation from snowfall and loss from melting. If glaciers advance, they accumulate more mass than they lose, and if they retreat, losses exceed gains. It follows that because nearly all glaciers worldwide are retreating, images of their retreat (as in Fig 1, which shows glaciers on the Norwegian island of Svalbard) capture the long-term global warming trend and are legitimate illustrations of climate change.

**Fig 2:**
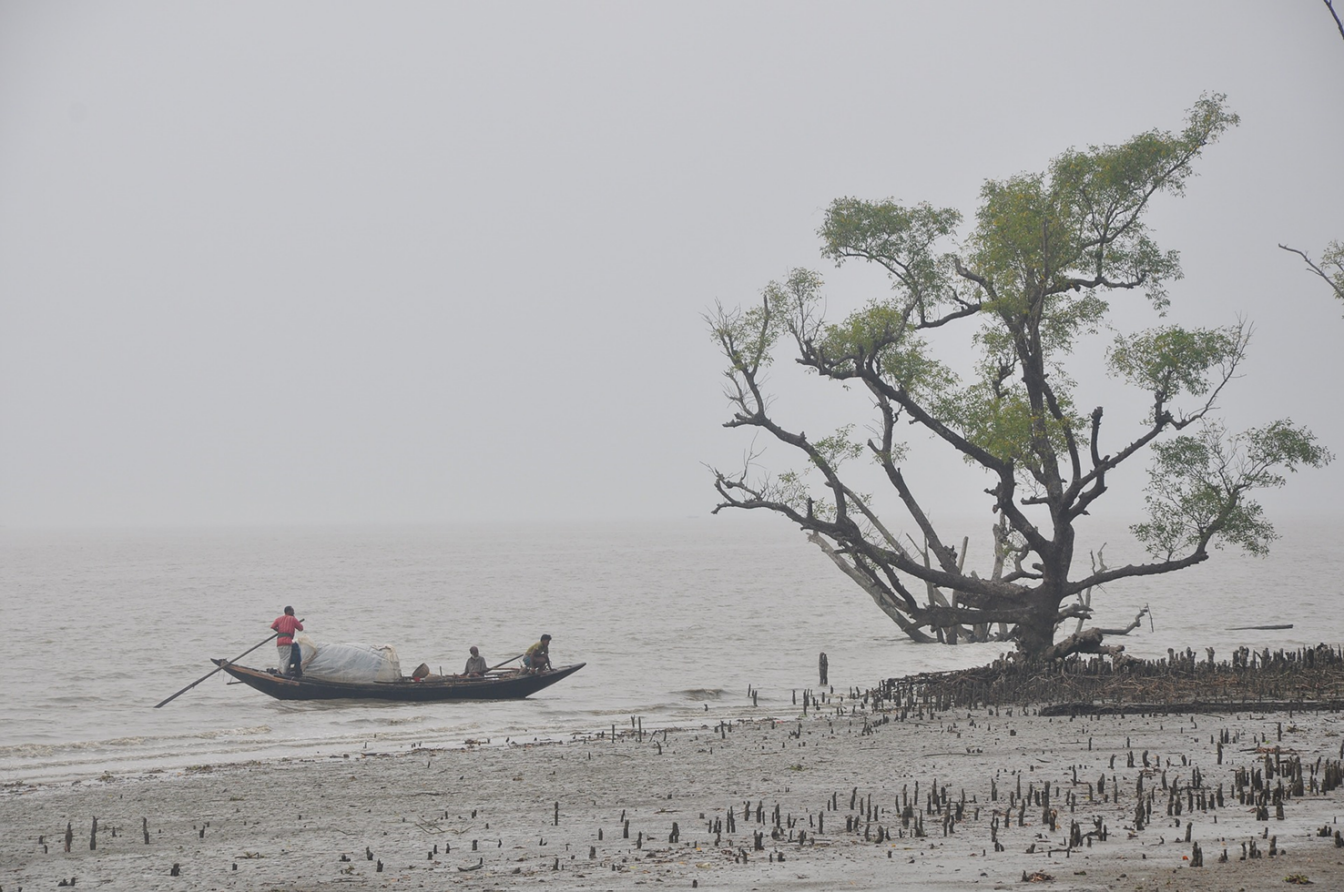
Coastal salination from sea level rise in Bangladesh. (Open Access license: https://pixabay.com/en/boat-sea-sundarban-tourism-nature-1511602/).

Sea level rise constitutes one of the least variable and statistically most detectable indicators of climate change. Unlike surface temperatures, which are subject to considerable fluctuations and changes in the rate of warming that can extend for a decade or more (e.g., [4]), sea level rise shows relatively little variability, and crucially, its effects are cumulative. Like glaciers, the mean sea level captures the long-term trend and is therefore a reliable and legitimate indicator of climate change. (Just like there are a small number of glaciers around the world that are not receding, there are some places on Earth where sea level rise may be slow or absent. This does not impair the legitimate role of sea level rise as an indicator of global warming, although it does rule out such cherry-picked instances as legitimate evidence against climate change.) It follows that stories or images that relate to the consequences of sea level rise—such as changes in soil salinity in coastal Bangladesh [19]—are legitimate illustrations of the consequences of climate change, as in Fig 2.

**Fig 3:**
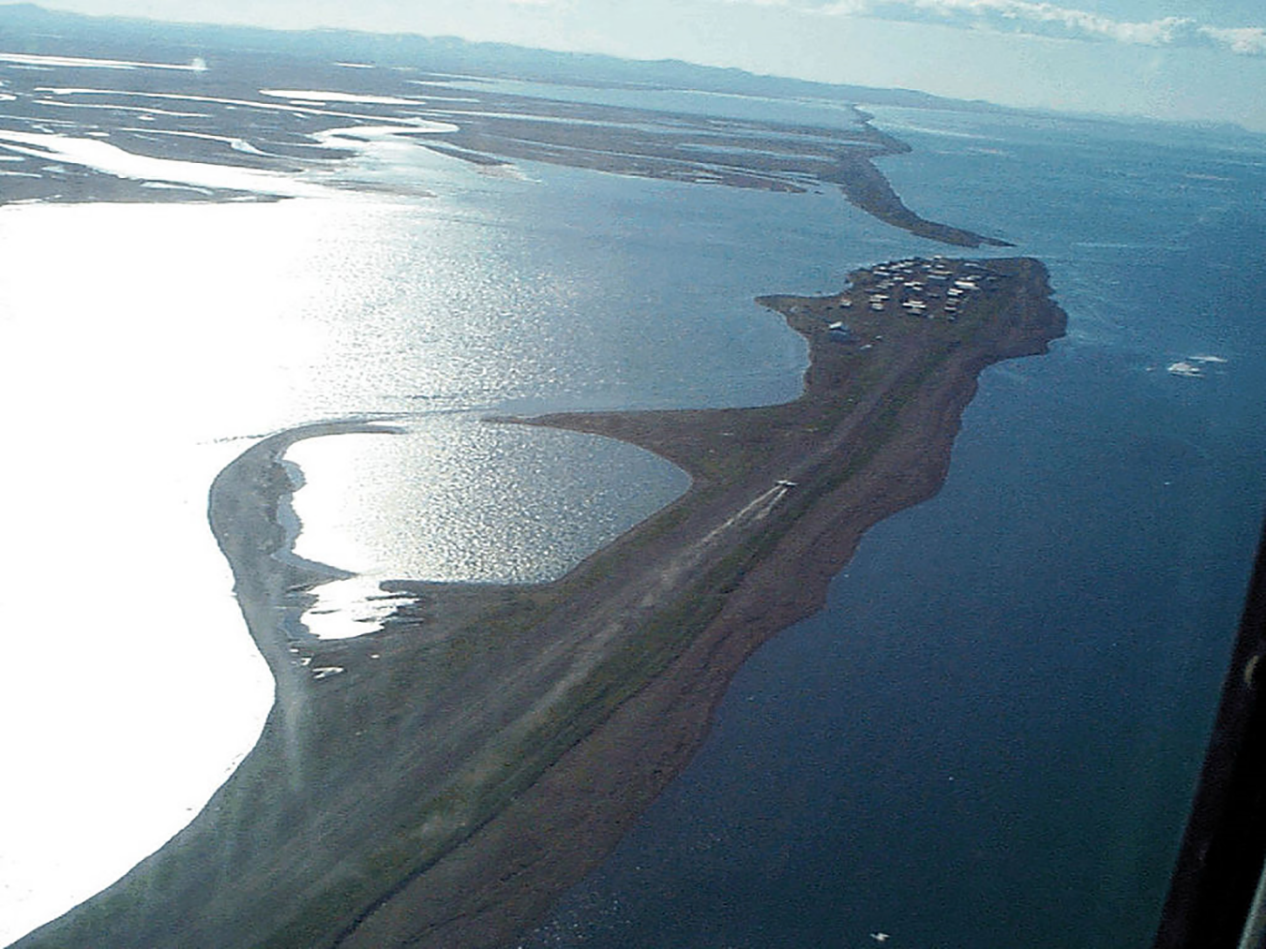
The Alaskan village of Kivalina is at risk from climate change. (Creative Commons license: https://commons.wikimedia.org/wiki/File%3AKivalina_Alaska_aerial_view.jpg).

Similarly, stories about the village of Kivalina in Alaska, which may have to relocate because of the changes resulting from climate change (e.g., https://toolkit.climate.gov/case-studies/relocating-kivalina), are legitimate illustrations and trigger points for affective engagement (Fig 3).

For biologists, long-term trends such as the clear changes in “distribution, phenology, community composition, abundance, demography and calcification across taxa and ocean basins” of marine organisms [20] or the clear pattern of phenological advance with warming [21] provide legitimate sources of illustration.

## Some pictures can tell a thousand words

A snowball represents weather. A retreating glacier represents climate change. This crucial distinction renders the former scientifically ludicrous and the latter a valid illustration of climate change that can legitimately be used for affective engagement with the public. Any image, anecdote, or story that captures a long-term trend that has been scientifically attributed to climate change can legitimately serve as a basis for raising public awareness. Snowballs, igloos, a cold winter in Egypt, or a starving polar bear do not satisfy those criteria. Biologists hoping to raise awareness of climate change risks to biodiversity should consider the evidence of how best to communicate climate change to ensure that they are not delivering counterproductive messages.

## References

[1] Joireman J., Truelove H. B., & Duell B. (2010). Effect of outdoor temperature, heat primes and anchoring on belief in global warming. Journal of Environmental Psychology, 30, 358–367. 10.1016/j.jenvp.2010.03.004

[2] Li Y., Johnson E. J., & Zaval L. (2011). Local warming: Daily temperature change influences belief in global warming. Psychological Science, 22, 454–459. 10.1177/0956797611400913 21372325

[3] Donner S., & McDaniels J. (2013). The influence of national temperature fluctuations on opinions about climate change in the U.S. since 1990. Climatic Change, 118, 537–550. 10.1007/s10584-012-0690-3

[4] Lewandowsky S., Risbey J. S., & Oreskes N. (2015). On the definition and identifiability of the alleged “hiatus” in global warming. Scientific Reports, 5, 16784 10.1038/srep16784 26597713 PMC4657026

[5] Harvey J. A., van den Berg D., Ellers J., Kampen R., Crowther T. W., Roessingh P., Verheggen B., Nuijten R. J. M., Post E., Lewandowsky S., Stirling I., Balgopal M., Amstrup S. C. & Mann M. E. (2017). Internet Blogs, Polar Bears, and Climate-Change Denial by Proxy. BioScience. 10.1093/biosci/bix133 29662248 PMC5894087

[6] Kahneman D. (2011). Thinking, fast and slow New York: Macmillan.

[7] Slovic P., Finucane M. L., Peters E., & MacGregor D. G. (2007). The affect heuristic. European Journal of Operational Research, 177, 1333–1352.

[8] Slovic P. (2000). The perception of risk. London: Earthscan.

[9] Loewenstein G. F., Weber E. U., Hsee C. K., & Welch E. (2001). Risk as feelings. Psychological Bulletin, 127, 267–286. 11316014 10.1037/0033-2909.127.2.267

[10] Weber E. U. (2006). Experience-based and description-based perceptions of long-term risk: Why globalwarming does not scare us (yet). Climatic Change, 77, 103–120.

[11] Lorenzoni I., Nicholson-Cole S., & Whitmarsh L. (2007). Barriers perceived to engaging with climate change among the UK public and their policy implications. Global Environmental Change, 17, 445–459.

[12] Whitmarsh L. (2011). Scepticism and uncertainty about climate change: Dimensions, determinants and change over time. Global Environmental Change, 21, 690–700. 10.1016/j.gloenvcha.2011.01.016

[13] Burke M., Ockwell D., & Whitmarsh L. (2018). Participatory arts and affective engagement with climate change: The missing link in achieving climate compatible behaviour change? Global Environmental Change, 49, 95–105. 10.1016/j.gloenvcha.2018.02.007

[14] O’Neill S. J., Boykoff M., Niemeyer S., & Day S. A. (2013). On the use of imagery for climate change engagement. Global Environmental Change, 23, 413–421.

[15] Wang S., Corner A., Chapman D. & Markowitz E. (2018). Public engagement with climate imagery in a changing digital landscape. WIREs Climate Change, 9, e509 10.1002/wcc.509

[16] Roosen L. J., Klöckner C. A., & Swim J. K. (2018). Visual art as a way to communicate climate change: a psychological perspective on climate change–related art. World Art, 8, 85–110.

[17] O’Neill S., & Nicholson-Cole S. (2009). “Fear won’t do it”: Promoting positive engagement with climate change through visual and iconic representations. Science Communication, 30, 355–379.

[18] Jacob T., Wahr J., Pfeffer W. T., & Swenson S. (2012). Recent contributions of glaciers and ice caps to sea level rise. Nature, 482, 514–518. 10.1038/nature10847 22318519

[19] Dasgupta S., Hossain M. M., Huq M., & Wheeler D. (2015). Climate change and soil salinity: The case of coastal Bangladesh. Ambio, 44, 815–826. 10.1007/s13280-015-0681-5 26152508 PMC4646857

[20] Poloczanska E. S., Brown C. J., Sydeman W. J., Kiessling W., Schoeman D. S., Moore P. J., Brander K., Bruno J. F., Buckley L. B., Burrows M. T., Duarte C. M., Halpern B. S., Holding J., Kappel C. V., O’Connor M. I., Pandolfi J. M., Parmesan C., Schwing F., Thompson S. A. & Richardson A. J. (2013). Global imprint of climate change on marine life. Nature Climate Change, 3, 919–925. 10.1038/nclimate1958

[21] Post E., Steinman B. A., & Mann M. E. (2018). Rates of phenological advance and warming have increased with latitude in the Northern Hemisphere over the past century. Scientific Reports, 8, 3297 10.1038/s41598-018-21611-7 29500377 PMC5834618

